# Coverage of non-receipt of cash transfer (Livelihood Empowerment Against Poverty) and associated factors among older persons in the Mampong Municipality, Ghana – a quantitative analysis

**DOI:** 10.1186/s12877-020-01786-3

**Published:** 2020-10-15

**Authors:** Doris Ottie-Boakye

**Affiliations:** Regional Institute for Population Studies, University of Ghana-Legon, Accra, Ghana

**Keywords:** Older persons, Cash transfer, LEAP, Mampong municipality

## Abstract

**Background:**

Social assistance in the form of cash transfer or in-kind has been recognised as a social protection strategy in many developing countries to tackle poverty and provide protection for individuals and households. Ghana’s cash grant programme, Livelihood Empowerment Against Poverty (LEAP), was introduced in 2008 to support selected households with vulnerable persons including older people 65 years and above, and persons with disabilities. This paper examined the coverage of non-receipt of LEAP, and the associated factors among older persons (65+ years) in the Mampong Municipality, Ghana.

**Methods:**

Data were extracted from the Ageing, Social Protection and Health Systems (ASPHS) survey carried out between September 2017 and October 2017 among older persons residing in LEAP-targeted communities. Data were analysed using descriptive and sequential logistic regression model techniques.

**Results:**

The mean age of respondents was 77.0 years and 62.3% were females. Rural residents constituted 59.0%. About 42.0% had no formal education and only 20.5% had no form of caregiving. Non-receipt of LEAP was 82.7% among study respondents. The fully adjusted model showed that being married (AOR = 3.406, CI 1.127–10.290), residing in an urban location (AOR = 3.855, CI 1.752–8.484), having attained primary level of education (AOR = 0.246, CI 0.094–0.642), and not residing in the same household with a primary caregiver (AOR = 6.088, CI 1.814–20.428) were significantly associated with non-receipt of cash grant among older persons.

**Conclusion:**

These results provide the first quantitative estimates of non-receipt coverage and its associated factors with the LEAP programme, which can inform the design of government policies related to cash transfers for older persons. The need for further research using different approaches to understand and explain the impact of cash grants on older persons’ well-being is crucial in strengthening old age social support care mechanisms in Ghana.

## Background

Social assistance in the form of a cash transfer (CT) or in-kind has been recognised as a social protection strategy in tackling poverty and providing financial protection for individuals and households. Though social protection was missing under the Millennium Development Goals (MDGs), it was recognised as a tool for alleviating poverty under the Sustainable Development Goals (SDGs). Social protection is influential in achieving a wider range of development goals such as health, social inclusion and poverty reduction [1]. It is estimated that at least 45.0% of the world’s population benefits from one social protection programme or the other. As of 2015, about 71.0% of the world’s population had not received the full range of benefits stemming from child and family benefits to old-age pensions [2]. The origin of social protection interventions is attributed to the western world where its role in meeting the MDGs has been well documented in countries such as the United States, Britain and Germany [3]. In developing countries, social protection has gradually found its place on the developmental agenda, and the African continent is not excluded from the rise and institutionalisation of social protection across the various continents in the world. According to the African Union (AU), social protection is defined as “a “package“ of policies and programmes with the aim of reducing poverty and vulnerability of large segments of the population” [4]. Social protection policies include cash transfers. Despite the gradual increase and extent of social protection programmes across continents, only a small portion of these interventions address the needs of older persons [3].

Globally, cash transfers as a form of social protection over the last few years have received attention as a poverty reduction strategy in many countries [5]. Cash transfers provide money to low-income families and households to help alleviate poverty and increase food consumption among such populations [6]. The Progresa progress programme in Mexico, Latin America was the first cash transfer programme to be designed and implemented. It was introduced as a national conditional cash transfer (CCT) program to reduce extreme poverty in Mexico. Currently, it is one of the most extensive CCT programs in the world [7]. Countries such as Brazil, Bolivia and South African have also implemented cash transfer programmes as key strategies to tackle poverty [8]. Cash transfer programmes, whether conditional (where recipients are expected to meet certain conditionalities) or unconditional (usually to persons such as older persons) have a positive impact on school attendance among children [9], reduction in HIV incidence among adolescents [10], uptake of nutrition interventions [11], children’s health improvements [1], and increased in household food consumption [6]. In Kenya, household food security is an immediate benefit of (conditional) cash transfers [12]. Among older persons, there is evidence of marked improvement in their dwelling characteristics and acquired household assets [13]. Old age is associated with age-related ill-health and disability, and in Ghana, elderly recipients of cash transfers utilized health services such as eye surgery and the required medication [14, 15]. Cash transfers have also been found to improve the social empowerment status, access to health services and economic security of persons with disabilities [16, 17].

Population ageing, which is the shift in the age structure of the world’s population towards the older age group affects both the developed and developing countries [18]. The reduction in fertility and mortality rates, rise in life expectancy, improvements in health technology, and advances in economic and industrial technology have resulted in population ageing [19]. Worldwide, it is estimated that a total of 1.5 billion older persons (over 60 years old) will be added to the world’s population by 2050 [20]. Eighty percent of older persons will be found in low and middle income countries (LMICs) [15, 21]. In Africa, the increase in the population of older persons coupled with the changing pattern in the care for older persons in the traditional family system may affect the social, cultural and support for older persons. Consequently, a cash transfer programme is crucial for older persons and the absence of cash transfers for older persons may have negative implications on their well-being. Urbanisation and the disintegration of family structures accompanied by the high cost of meeting quality and appropriate health care services predispose older populations to diseases and ill-health challenges [22]. Poverty affects older persons adversely. The risk of poverty among the elderly is significant relative to younger adult persons [23]. Older populations often fall into the poorest population groups in most developing countries. This has been attributed to the inequalities they experienced early in life [24]. According to the International Labour Organisation (ILO), older persons continue to depend on family support arrangements [2]. Non-contributory pensions such as cash transfers have been implemented in developing countries to reduce poverty in old age [25]. This intervention is not only crucial into meeting the needs of older persons, but it also helps in achieving the Sustainable Development Goals to reduce all forms of poverty (Goal 1), ensure healthy lives and the promotion of well-being (Goal 3), and achieve gender equality (Goal 5) [24].

There are diverse actors implementing cash transfer programmes for the elderly ranging from the traditional social institutions to state actors, Non-Governmental Organisations (NGOs), international NGOs (INGOs) and bilateral donors [5]. The impact of cash transfers begins with the recipient, and then expands to the household, wider community, and eventually the country [26]. However, some studies have pointed out errors of inclusion and exclusion in targeting recipients for cash transfers [27–30]. For instance, in most cases, older adults who are not poor are included while excluding poor prime-aged adults [27]. In the context of these arguments, this paper explores the coverage of non-participation in the cash grant programme (Livelihood Empowerment Against Poverty (LEAP)), and the associated factors among older persons (65+ years) in the Mampong Municipality, Ashanti region. This study provides the first quantitative analysis of the coverage of non-receipt, and associated factors with the LEAP programme in LEAP-targeted communities in the Ashanti region of Ghana. In doing so, this paper contributes to the limited body of evidence on non-receipt of cash transfers among older persons in the Ghanaian context.

### Cash transfer programme in Ghana

Ghana has employed several programmes with social protection prospects. The National Social Protection Strategy (NSPS) was developed in 2007. The Government of Ghana’s cash grant programme, LEAP was piloted in 2008 in selected districts across the country, including the Mampong Municipality. Upon the revision of the NSPS document in 2012, a Social Protection Rationalization Study was carried out in 2013 to develop a holistic National Social Protection Policy. In 2014, the policy was developed and approved by Cabinet. In 2016, the Ghana National Social Protection Policy (GNSPP) was launched, making provision for an effective, efficient and coherent delivery framework for social protection [23]. Further, the framework is a demonstration of the country’s endorsement of the SDGs [23]. The Government of Ghana’s cash transfer programme, LEAP, is a social cash transfer programme which provides cash and health insurance to impoverished households [31]. LEAP covers older persons aged ≥65 years without any form of support, severely disabled without productive capacity, orphaned and vulnerable children (OVC), and indigent households with pregnant women or mothers with infants [32]. For older persons, the LEAP programme is an unconditional cash transfer for those ≥65 years old with no productive capacity due to poverty, vulnerability and exclusion [33].

The programme currently reaches 213,044 beneficiary households as of April 2018, covering 254 districts across Ghana [32]. Monthly transfers to LEAP beneficiaries ranged from GH 64.00 to GH 106.00 per household per month (approximately US$ 11.56–19.14 based on the exchange rate of March 2020) depending on the number of eligible individual members per household. Beneficiaries are selected at the household level cascaded from the national, regional, district and community levels [34]. These are done through a nationally generated poverty map and rankings obtained from the Ghana Statistical Service [34], a body mandated by law to produce statistics for the country. Proxy Means Test (PMT) questionnaire is administered to a household member(s) and is/are qualified based on the PMT formula and pre-defined threshold or cut-off points [34]. Beneficiary households are then enrolled in the programme.

## This present study

Evidence relating to factors associated with older persons’ non-receipt of cash transfers is scarce. Most studies focused their analysis on only beneficiaries, hence, missing out on the extent of coverage and the associated factors concerning non-participation in cash transfers among older persons [30, 35–37]. Much of the earlier work concentrated on the importance and impacts of these transfer programmes on old age and ageing in developing countries [25, 26, 37–42]. Though there have been studies on older persons’ participation in cash transfers in Ghana [36, 41, 42], there is limited available data on the coverage of non-receipt of cash transfer and the associated factors among older persons in LEAP-targeted communities in Ghana. This study seeks to address these identified gaps in the literature by making a modest contribution to the extent of non-participation of older persons in cash transfer programmes in the context of Ghana.

## Methods

### Study setting

This present study used data collected in the Mampong Municipality. At the time of the study, the municipality was one of the 20 administrative districts in the region. It was formed from the then Sekyere West District by the Legislative Instrument (L.I.) 1908 [43]. The municipality is divided into seven zonal councils and is managed by the Municipal Assembly. The assembly directs and oversees the daily administrative maintenance in the provision of services as well as policy formulation and implementation for its population [44]. Though other ethnic groups from across Ghana and the sub-region are located in the municipality, Akan is the dominant ethnic group [43]. The 2010 Population and Housing Census (PHC) showed an increase in the population of the municipality from 78,056 in 2000 to 88,051 in 2010 [43]. This represents a 12.8% increase, and a 1.3% growth rate per year from 2000 to 2010. The 2017 performance review report of the Municipal Health Directorate estimated that the catchment population served was 99, 924 [45]. Females and males constituted 51.6 and 48.4% of the total population respectively. The municipality’s demographic profile showed a youthful population with a vast proportion of the youth being below 15 years of age [43]. The municipality is more rural (54.7%) than urban (45.3%) and the economy is agriculture-based. About 61.0% of households are engaged in agriculture. The dominant form of agriculture is crop farming. Whereas 78.6% of households in rural communities are engaged in agriculture, this is 42.4% among urban dwellers. The majority are engaged in the private informal sector (87.6%), and this is higher for females (90.8%) than males (84.2%). Residents in the municipality benefits from pro-poor social interventions like the Livelihood Empowerment Against Poverty [44]. The LEAP covers beneficiaries such as elderly persons aged ≥65 years who are extremely poor, caregivers of orphans and vulnerable children, and persons with disabilities without productive capacity [46]. By the end of the fourth quarter in 2017, the municipality had ten communities with a total of 825 households as LEAP recipients [46].

The socio-cultural context of the municipality showed an average household size of 4.5 with 61.0% being headed by males [43]. Social intervention programmes such as LEAP and NHIS are sometimes targeted at the household level [43]. This is because households serve as the foundation in the study of social welfare [47]. Marriage is regarded as social status, a responsibility, trust and achievement [48]. One-third of those aged 12 years and above were married; this is higher among females (40.1%) than males (38.5%). Among older persons, about half were married; this is higher among males (73.3%) than their female counterparts (33.1%).

The municipality was selected for the study due to its inclusion in the LEAP pilot programme in 2008, and the fairer rural-urban population distribution in the Ashanti region. In effect, the LEAP programme uses cash transfers as incentives to lessen the burden of hardship among older populations [49], access medication or pay for transportation to health facilities [12], and improve the health of ageing adults [35]. At the end of 2017, the municipal had close to 900 beneficiary households [46]. The country also has a National Policy on Ageing to ensure that older persons participate actively in society and development. However, this document does not address the social protection needs of older persons or develop appropriate social protection policies solely for the elderly.

### Data and sample

Data were extracted from the Ageing, Social Protection and Health Systems (ASPHS) survey conducted between September 2017 and October 2017 among older persons aged ≥60 years residing in eight LEAP-targeted communities in Mampong Municipality. A household population register was generated from household listing exercise in twelve enumeration areas in the eight communities. ASPHS survey data covered 400 non-institutionalized older persons (60 years and above) sampled through stratified (location and sex) and simple random sampling techniques. The 400 respondents were sampled using Open Source calculator—SSPropor formula from OpenEpi Version 3.01 [50],

$$ n= deff\times \frac{N\hat{p}\hat{q}}{\frac{d^2}{1.96^2}\left(N-1\right)+\hat{p}\hat{q}} $$

where.

n = sample size.

deff = design effect = 1.

N = elderly population size in Mampong Municipality = 6420 [43].

p = % frequency of access to universal basic healthcare for the elderly = 57.0% ± 5.

q = 1- p.

d = desired absolute precision/absolute level of precision = 5.0%.

Therefore,

n = [1*6420(0.57)(1–0.57)]/[((0.05)^2^/(1.96)^2^ *(6420–1) + 0.57*(1–0.57)].

*n* = 356.

To make room for non-response and maintain statistical power, the sample size was rounded up to 400 to account for non-response and maintain statistical power. To avoid biases, participating in the study was restricted to one older person in a household. Participation was based on the “willingness to participate” method in households with more than one older person. Older persons were the primary respondents, and data were collected at both the household and individual level. However, at the household level, information such as dwelling characteristics, household possessions, and food security was elicited from older persons or eligible household members. Data were collected using structured questionnaires embedded in an electronic device and were administered face-to-face by four trained research assistants. The questionnaires covered demographic, socioeconomic, behavioural and lifestyle risks, health and health behaviours, and work history. Other information collected covered disability and social protection participation. The estimated burden of time for each questionnaire was about 69 min. Though the data collection tools were developed in English, they were accurately translated into the local language to collect responses from study participants. Responses were retranslated into English after data collection. The data collection process was monitored and supervised by the author.

Ghana’ cash grant programme targets those 65 years and older, especially those without productive capacity. Hence, the analysis was based on responses from 313 respondents aged 65 years and above.

### Measures

#### Outcome variable

Coverage of cash transfer was measured as a dichotomous variable indicating ‘non-receipt’ or ‘receipt’ of cash transfer at the time of the data collection. Study respondents were classified as ‘non-receipt’ or ‘receipt’ of cash transfer based on the verification of cards. Respondents with LEAP card or who self-reported as recipients were classified as being ‘recipient’ while those without cards or could not report being recipients of the cash grant were categorised as ‘non-receipt’ of the programme.

#### Predictor variables

The associated factors were analysed in five areas: demographic, socio-economic, lifestyle risk factors, living arrangement and health-related factors. The socio-demographic variables were age in years (1 = 65–69, 2 = 70–74, 3 = 75–79, 4 = 80–84, 5 = 85 or above), sex (1 = Female, 2 = Male), marital status (1 = married, 2 = not married), and location (1 = rural, 2 = urban). The socio-economic variables were education level attained (1 = no formal education, 2 = Primary education, 3 = Middle school, 4 = Secondary and above), occupation (1 = no occupation, 2 = Agriculture, 3 = Non-agriculture), household wealth index (1 = Poor, 2 = Middle, 3 = Rich); and household food security (0 = Not food secured, 1 = Food secured). Life style risk factors were consumption of tobacco (1 = Ever smoked, 2 = Never smoked) and consumption of alcohol (1 = Ever consumed, 2 = Never consumed). Self-rated health status (1 = Bad, 2 = Moderate, 3 = Good), and having non-communicable diseases (NCDs) (1 = Yes, 0 = No) were the health-related variables. Marital status were dichotomized [36]. Household wealth index was generated based on household living assets and possessions [51]. Household food security was measured based on the availability and access to food by households of study respondents within the last 30 days preceding the survey [52, 53].

#### Analytical framework

Descriptive analyses were performed to describe the background characteristics of the study sample. Again, the coverage of non-receipt of cash transfer was also examined. Additionally, sequential logistic regression models were employed to predict the variables that were associated with non-receipt of the cash transfer programme. Sequential logistic regression technique was employed to demonstrate the predictive abilities of each covariate. Further, it also specifies the order with which these predictor variables enter the models [54]. Five different sets of models were established to estimate these predictors of non-receipt of the cash transfer programme. Model 1 consisted of demographic variables. Model 2 comprised socio-economic variables in addition to all variables in Model 1. Model 3 constituted all variables in Model 2 plus lifestyle risk factors. Model 4 constituted all variables in Model 3 plus living arrangements variables. Model 5 (full Model) constituted all variables in Model 4 in addition to health-related variables. A significant level of 0.05 and an odds ratio (OR) with 95% confidence interval (CI) were reported. All analyses were conducted with STATA version 14.0.

## Results

### Descriptive statistics

#### Background characteristics of study respondents

Table 1 presents the background characteristics of the respondents. The mean age was 77.0 years (SD ± 0.561). About 62.0% were females. Close to half (49.5%) were widowed. About 59% reside in rural communities. Forty-two percent had no formal education and almost half were not engaged in any economic activity. One-third of participants were from poor, middle and rich households respectively. Sixty-seven percent were from households with food security. Eighty-three percent did not have a history of smoking while 62.3% were lifetime abstainers of alcohol consumption. Whereas 34.2% live alone, 20.5% had no form of caregiving. Averagely, one-third of the respondents perceived their health status to be good, moderate or bad respectively. A proportion of 80.2 had or suffered from at least one form of NCD such as stroke, hypertension or diabetes.

#### Coverage of non-receipt of cash transfer (LEAP) among study respondents

Figure 1.1 displays the non-receipt of Cash Transfer among the study respondents. Non-receipt of cash transfer, LEAP was 82.7%, and only 17.3% were recipients of the cash transfer.

### Multiple logistic regression models

#### Factors associated with non-receipt of cash transfer Programme

Table 2 presents results on factors associated with non-receipt of cash transfer (LEAP) among older persons. In Model 1, the study found that respondents residing in urban centres were 3 times as likely as their counterparts in rural areas to be non-recipients of the cash grant (Adjusted Odds Ratio (AOR) = 3.332; CI 1.643–6.755).

In Model 2, location and education level were statistically significant after the introduction of socio-economic factors. Those who reside in urban centres were 3.5 times as likely as their counterparts in rural communities to be non-recipients of the cash grant. The model further showed that attaining a primary level of education had 0.66 lower odds of non-participation in the cash transfer programme relative to those with no education. These were statistically significant.

With the introduction of lifestyle risk factors in Model 3, results from the study showed that urban respondents were four times as likely as rural dwellers to be non-recipients of cash transfer (AOR = 3.419; CI 1.623–7.203). Also, respondents who had attained primary level of education had 74.0% lower odds of being non-cash transfer recipients relative to those with no formal education, and this was significantly significant (AOR =0.339; CI 0.137–0.838). This implies that despite the introduction of lifestyle risk factors in Model 3, location and education level attained still explain non-receipt of cash transfer among study respondents.

In Model 4, the introduction of living arrangement factors significantly showed that study respondents who were married were 3.5 times as likely as those not married to be non-recipients of cash transfer (AOR = 3.492; CI 1.156–10.546). Significantly, respondents without primary caregiver were six times as likely as those with caregivers in separate households to be non-recipients of the cash grant (AOR = 6.089; CI 1.820–20.372). Location and education level attained were still statistically significant even after introducing living arrangement factors. This means respondents’ location (urban/ rural) and the educational level attained contribute to non-participation in cash transfer programmes.

Finally, with Model 5, respondents who are married (AOR = 3.406, CI 1.127–10.290), residing in urban communities (AOR = 3.855, CI 1752–8.484), having no primary caregiver (AOR = 6.088, CI 1.814–20.428) were significantly three times, about four times and six times as likely as their counterparts to be non-cash transfer recipients relative to their respective counterparts. On the other hand, respondents with a primary level of education (AOR = 0.246, CI 0.094–0.642) were significantly less likely to be non-recipients of cash transfer. This means the later are more likely to be cash transfer recipients. The final model, Model 5 with the introduction of the health-related factors such as self-rated health status and having NCD showed that marital status, location, education and having a primary caregiver were strong factors associated with non-receipt of cash transfer among older persons.

## Discussion

The purpose of this study was to explore the coverage of non-participation in the cash grant programme, LEAP, and the associated factors among older persons in the Mampong Municipality in the Ashanti region of Ghana. The study found that majority of older persons residing in LEAP-targeted communities were non-recipients of the cash transfer. Although there is a rise in cash transfers as a form of formal social protection mechanisms in many developing countries [55], and the eroding of extended family support systems in most developing countries [56, 57], majority of older persons are non-recipients of the cash grant. The coverage gap in cash transfer has been found to exist among older persons, and this has been attributed to implementation or take-up problems [58]. Interestingly, the National Social Protection Policy reported that there is inadequate coverage of cash transfer, LEAP, which provides social assistance to older persons [23]. There is evidence that the contribution of cash transfer as social protection to the survival and livelihood of beneficiaries, especially older persons are enormous [59–61]. In most sub-Saharan African (SSA) countries, there is low coverage of contributed-based pension schemes [62]. Cash transfer programmes such as LEAP serve as a stop-gap in fighting poverty for older persons. Nevertheless, there is evidence that targeting older persons in cash transfers compared to other target populations such as children and women of child-bearing age [8, 63, 64] is inadequate [64].

It was observed that marital status positively affected non-receipt of cash transfer, especially those who were in marital unions. Unmarried older persons were more likely to be recipients of cash transfers relative to those who were married. This was consistent in two of the models. Marriage is regarded as social status, a responsibility, trust and achievement [48]. About two-thirds of the study respondents were not married. This could be due to being widowed, divorced or never married. Often, many older persons are typically widowed [19]. Some form of social protection and social capital and network could be derived from marriage for older persons through spousal and relation support [19]. There is evidence that older persons who are not married are potentially associated with food insecurity [65–68], and this has implications for poverty. Hence, the need for cash transfers.

The findings from this study highlight the importance of location in cash transfer participation in Ghana. This effect did not vary across the five models. The analysis showed that older persons are more likely to reside in rural areas than in urban centres. This finding is supported by earlier studies [19, 69]. Findings from this study showed that about half of the participants had no occupation. Those who are employed are engaged in small scale agriculture where access to income and social security are often limited. Many of them have or had suffered from one form of non-communicable diseases such as stroke, hypertension and diabetes. The country’s adult hypertension prevalence ranges from 19.0 to 48.0% [70, 71], and the analysis showed that most participants have or have had one form of non-communicable diseases such as stroke, hypertension and diabetes. Hypertension prevalence was higher in urban areas compared to rural localities [72]. In poor urban communities, females were found to be more hypertensive than males [73]. From nationally representative data on older adults ≥50 years in Ghana, the author found rural residents to be twice as likely as those in urban areas to have a chronic non-communicable condition. However, there was no variation by sex [71]. The prevalence of diabetes and stroke among older adults ≥50 years in Ghana is 7.0 and 4.9% respectively [71]. Hence, given the criteria for participating in LEAP being 65 years and older without productive capacity may have contributed to this rural-urban differentials. Having urban older persons less likely to be cash grant recipients have implications for heightening intra urban inequalities. In contrast, studies have reported that urban dwellers especially workers in regular wage employment benefit from social protection such as social assistance as compared to the rural majority than those in informal urban settlements in developing countries [74]. The need to further explore this variation in urban slums dwellers, particularly, among older persons is not only crucial for human existence but policy reforms. Contrarily, other studies have asserted that fending for one’s self was one of the challenges among rural older persons in Ghana, attributing it to the lack of pension schemes for older persons [75].

Education also negatively affected the non-receipt of LEAP. Having attained primary education consistently increased the effect of being a cash transfer recipient in four models. Further analysis of the results showed some level of association between education level attained and the presence of the primary caregiver in the same household. Participants with no formal education were more likely to reside in separate households with their primary caregiver. The presence of a primary caregiver was associated with participating in cash transfers in this study. Furthermore, in Ghana, the main selection process for LEAP has been through household using the proxy means test [31]. This methodology has been reported to be associated with a very small proportion of potential beneficiaries [30, 76]. Nonetheless, a correlation between social cash transfer programmes and education among beneficiaries in developing countries like Malawi has also been reported [30]. The authors reported that beneficiaries tend to have very little or no formal education [30]. In a qualitative study in the region of the study area, poverty and multiple deprivations were found to be associated with old age [77]. The author further attributed this to the low levels of education among older persons [77]. Hence, cash transfers ensure access to basic needs such as food, health and decent livelihoods.

Previous studies show that the growing complexity of the living arrangement of children of older persons will result in weaker family ties. This will lessen their support for ageing parents [15]. This study revealed that about a third of the respondents live alone and one-fifth have no primary caregiver. Although a substantial proportion lives alone, earlier studies had found lower proportions [78, 79]. But, caregiving (informal) is common in most developing countries, including Ghana [80]. Those without a primary caregiver were more likely to be non-recipients of the cash transfers by six folds relative to those with a caregiver in separate households. This implies that the presence of a primary caregiver has a positive influence on participating in cash grant programmes. It has been reported in the United States that caregivers (informal carers) often act as mediators in ensuring older persons access social services such as health [81], for which cash grant programmes are not exempted. In the effort to improve the coverage of cash transfers among older persons, there is a need for policymakers and implementers to build a bridge between carers and the success of grant programmes.

The study further observed that informal social protection such as family or informal caregiving complements formal social protection like cash transfers. Some older persons depend on the assistance of non-state actors such as the family system. In the effort to improve the overall quality of life for older persons, there is a need for policymakers and practitioners to strengthen the relationship between formal and informal social protection programmes. This may be boosted through community engagements, incorporating local leadership in the care for older persons devoid of political interference, intensification of education and sensitisation on old age, the ageing process and the associated challenges.

Studies on social pensions particularly for older persons have cited age (60 or 65 years and above), poverty, disability and unemployment as the selection criteria in most developing countries including Ghana [23, 82, 83, 84]. Exploring evidence from Ghana’s LEAP programme in the Nadowli-Kaleo district in the Upper West region using a qualitative exploratory research design, the authors found that more women compared to men receive LEAP benefits [42]. This was attributed to poverty being pronounced among women than men especially in Africa, where women suffer from restrictions in choices and opportunities relative to men [41]. Also, in rural Malawi, beneficiaries were found to be women and persons with very little or no formal education [30]. However, this study found no association between factors like age, sex, occupation, and household wealth index, and non-receipt of cash transfers among older persons. Findings from this study, therefore, could be attributed to the targeting methodology that measures household-level characteristics but not that of older persons such as the nature of remittances and levels of ill-health [30, 76]. Other studies have also underscored the existing political and institutional-related weaknesses characterising the exclusion and inclusion errors in older persons’ receipt in cash grant programmes [40, 85]. For instance, older persons with direct connections to local government officials are more likely to be beneficiaries compared to poor older persons without these connections [40].

### Limitations

Some limitations may have influenced the results of this study, and there is a need to point them out. Firstly, the cross-sectional nature of the study does not allow the causality of findings to be determined. Secondly, the study was carried out in LEAP-targeted communities which may limit the generalizability of findings. This is because the findings may vary among older persons who do not reside in LEAP-targeted communities. Thirdly, this study did not explore the experiences and perceptions about cash transfers among study participants. Future studies should utilise other research approaches in exploring the perceptions and experiences of cash transfers to complement the flaws in quantitative research methodology. Lastly, future studies should examine institutional-related factors that may influence older persons’ participation in cash transfer programmes which was not addressed in this current study.

## Conclusions

While this study quantitatively investigated the coverage of non-receipt of cash transfers, further research is required regarding perceptions, experiences and impact on wellbeing, especially in the study setting. Most participants were found to be non-recipients of the cash grant. This study recommends the need for a non-contributory universal old-age pension scheme compared to the target setting cash transfer programme for older persons. Furthermore, the study investigated the associated factors at the microsystem such as intrapersonal and household levels. The study findings underscore the relevance of demographic, socio-economic and living arrangements for non-receipt of cash transfer in Ghana, particularly in the study setting. Lifestyle risk factors and health-related factors had no effects on non-receipt of cash transfers. This indicates that households with older persons adequately participate in the cash transfer programme especially, in rural localities. This emphasises on the significance of individual and household factors for Ghana’s cash grant programme. This calls for the consideration of individual characteristics of older persons in targeting programmes such as cash grants taking into account the inequalities that exist within households. Currently, the commonly used eligibility criteria in participating in the cash grant programme focus on household-level characteristics resulting in non-receipt among older persons. Hence, further studies evaluating the performance of Ghana’ cash transfer programme targeting older persons at the individual level is in line with the country’s revised 2010 National Ageing Policy calling for the need to strengthen social protection schemes for older persons. This will not only support dignified ageing among older persons, but it will also ensure they have full economic and social participation in society. This study also provides the platform for further research regarding the macro-system exploring the various actors such as community, institutional and at the national policy level involvements in the provision of cash transfers particularly for older persons in Ghana.

## Supplementary information

**Additional file 1: Figure 1:** Non-receipt of cash transfer (LEAP) among study respondents.

**Additional file 2: Table 1:** Background characteristics of the study respondents.

**Additional file 3: Table 2:** Sequential logistic regression results on the factors associated with non-receipt of cash transfer among older persons.

## Data Availability

The data that support the findings of this study are available from the corresponding author, [DOB], upon reasonable request.

## Abbreviations

AOR: Adjusted Odds Ratio
ASPHS: Ageing, Social Protection and Health Systems
AU: African Union
CI: Confidence Interval
CT: Cash Transfer
CCT: Conditional Cash Transfer
DSW: Department of Social Welfare
ECH: Ethics Committee for Humanities
ERC: Ethics Review Committee
GHS: Ghana Health Service
GNSPP: Ghana National Social Protection Policy
GoG: Government of Ghana
GSS: Ghana Statistical Service
LI: Legislative Instrument
ILO: International Labour Organisation
INGOs: International Non-Governmental Organizations
ISSER: Institute of Statistical, Social and Economic Research
LEAP: Livelihood Empowerment Against Poverty
LMIC: Low and Middle-Income Countries
MDGs: Millennium Development Goals
MoGCSP: Ministry of Gender, Children and Social Protection
NCDs: Non-communicable Diseases
NGEC: National Gender and Equality Commission
NGOs: Non-Governmental Organizations
NSPS: National Social Protection Strategy
OR: Odds Ratio
OVC: Orphaned and Vulnerable Children
PHC: Population and Housing Census
PMT: Proxy Means Test
SDGs: Sustainable Development Goals
SSA: Sub-Saharan African
UN: United Nations
