# Supplementary material for: Coverage of non-receipt of cash transfer (Livelihood Empowerment Against Poverty) and associated factors among older persons in the Mampong Municipality, Ghana – a quantitative analysis
Source: BMC Geriatr. 2020 Oct 15;20:406. doi: 10.1186/s12877-020-01786-3 (PMC7566032; doi:10.1186/s12877-020-01786-3)
Supplement: Supplementary file 1 — Additional file 1: Figure 1: Non-receipt of cash transfer (LEAP) among study respondents. [file 12877_2020_1786_MOESM1_ESM.docx]

Figure 1.1: Non-receipt of cash transfer (LEAP) among study respondents
