# Supplementary material for: Coverage of non-receipt of cash transfer (Livelihood Empowerment Against Poverty) and associated factors among older persons in the Mampong Municipality, Ghana – a quantitative analysis
Source: BMC Geriatr. 2020 Oct 15;20:406. doi: 10.1186/s12877-020-01786-3 (PMC7566032; doi:10.1186/s12877-020-01786-3)
Supplement: Supplementary file 2 — Additional file 2: Table 1: Background characteristics of the study respondents. [file 12877_2020_1786_MOESM2_ESM.docx]

**Table 1: Background characteristics of the study respondents**

**Variable Mean Standard deviation**

**Age** 77.0 0.561

***Socio-demographic characteristics***

**Variable Category %**

**Percentage N=313**

**Age group**

65-69 23.3 73

70-74 21.4 67

75-79 20.8 65

80-84 16.6 52

85+ 17.9 56

**Sex**

Female 62.3 195

Male 37.7 118

**Marital status**

Married 31.3 98

Not married 68.7 215

**Location**

Rural 58.8 184

Urban 41.2 129

***Socio-economic characteristics***

**Highest level of education attained**

No education 41.8 131

Primary 16.3 51

Middle 33.9 106

Secondary and above 8.0 25

**Occupation**

No occupation 49.2 154

Agriculture 37.4 117

Non-agriculture 13.4 42

**Household wealth index**

Poor 36.7 115

Middle 31.3 98

Rich 32.0 100

**Household food secured**

Food secured 66.8 209

Not food secured 33.2 104

***Lifestyle risk factors***

**Smoking status**

Ever smoked 17.3 54

Never smoker 82.7 259

**Alcohol consumption status**

Ever consumed 37.7 118

Never consumed 62.3 195

***Living arrangements***

**Household size**

Alone 34.2 107

2-3 members 38.3 120

4 or more members 27.2 85

Unknown 0.3 1

**Having a primary caregiver**

No 20.5 64

Same household 37.7 118

Separate household 41.8 131

***Health-related characteristics***

**Self-rated health status**

Good 34.8 109

Moderate 33.2 104

Bad 32.0 100

**Having NCD**

Yes 80.2 251

No 19.8 62

*Source*: Computed from (ASPHS) survey data, September 2017 – October 2017
