# Supplementary material for: Coverage of non-receipt of cash transfer (Livelihood Empowerment Against Poverty) and associated factors among older persons in the Mampong Municipality, Ghana – a quantitative analysis
Source: BMC Geriatr. 2020 Oct 15;20:406. doi: 10.1186/s12877-020-01786-3 (PMC7566032; doi:10.1186/s12877-020-01786-3)
Supplement: Supplementary file 3 — Additional file 3: Table 2: Sequential logistic regression results on the factors associated with non-receipt of cash transfer among older persons. [file 12877_2020_1786_MOESM3_ESM.docx]

**Table 2 Sequential logistic regression results on the factors associated with non-receipt of cash transfer among older persons**

**Variable Model 1 Model 2 Model 3 Model 4 Full model**

AOR 95%CI AOR 95%CI AOR 95%CI AOR 95%CI AOR 95%CI

***Socio-demographic***

**Age (years)**

65-69 1 1 1 1 1

70-74 0.586(0.223-1.540) 0.702(0.250-1.974) 0.698(0.247-1.975) 0.699 (0.235-2.077) 0.666(0.221-2.006)

75-79 0.976(0.354-2.694) 1.106(0.377-3.250) 1.089(0.370-3.207) 1.324 (0.429-4.087) 1.226(0.387-3.884)

80-84 0.513(0.189-1.392) 0.584(0.195-1.750) 0.560(0.186-1.690) 0.706 (0.217-2.295) 0.655(0.194-2.219)

85+ 0.438(0.165-1.158) 0.407(0.130-1.270) 0.379(0.119-1.208) 0.438 (0.130-1.481) 0.393(0.112-1.375)

**Sex**

Female 1 1 1 1 1

Male 1.174(0.507-2.718) 1.210(0.472-3.100) 1.046(0.376-2.911) 0.769 (0.257-2.303) 0.792(0.261-2.403)

**Marital status**

Not married 1 1 1 1 1

Married 2.330(0.893-6.080) 2.469(0.909-6.701) 2.580(0.948-7.024) 3.492*(1.156-10.546) 3.406*(1.127-10.290)

**Location**

Rural 1 1 1 1 1

Urban 3.332*(1.643-6.755) 3.516*(1.675-7.378) 3.419*(1.623-7.203) 4.017*(1.831-8.810) 3.855*(1.752-8.484)

***Socio-economic***

**Education attained**

No education 1 1 1 1

Primary 0.337*(0.136-0.835) 0.339*(0.137-0.838) 0.263*(0.102-0.677) 0.246*(0.094-0.642)

Middle 0.960 (0.390-2.364) 0.953(0.385-2.358) 0.883 (0.343-2.274) 0.841(0.322-2.195)

Secondary+ 0.760 (0.167-3.450) 0.751(0.163-3.457) 0.651 (0.133-3.176) 0.607(0.120-3.056)

**Occupation**

No occupation 1 1 1 1

Agriculture 1.520 (0.702-3.294) 1.509(0.694-3.278) 1.383 (0.598-1.934) 1.341(0.569-3.160)

Non-agriculture 1.546 (0.520-4.591) 1.549(0.521-4.605) 1.641 (0.533-5.050) 1.630(0.526-5.055)

**Household wealth index**

Poor 1 1 1 1

Middle 0.853 (0.382-1.907) 0.862(0.385-1.930) 0.831 (0.357-1.934) 0.891(0.379-2.097)

Rich 0.775 (0.352-1.704) 0.779(0.355-1.713) 0.764 (0.330-1.766) 0.782(0.337-1.816)

**Household food secured**

No 1 1 1 1

Yes 1.347 (0.692-2.625) 1.371(0.697-2.695) 1.679 (0.786-3.587) 1.806(0.812-4.018)

***Lifestyle risk factors***

**Smoking status**

Never smoked 1 1 1

Ever smoked 1.860(0.581-5.953) 2.021 (0.621-6.573) 2.139(0.653-7.006)

**Alcohol consumption status**

Ever consumed 1 1 1

Never consumed 1.203(0.574-2.519) 1.068 (0.486-2.349) 1.110(0.504-2.445)

***Living arrangements***

**Household size**

Alone 1 1

2-3 members 0.751 (0.314-1.796) 0.726(0.301-1.752)

≥4 1.098 (0.402-2.993) 1.065(0.387-2.926)

**Having a primary caregiver**

Separate household 1 1

No primary caregiver 6.089*(1.820-20.372) 6.088*(1.814-20.428)

Same household 0.992 (0.445-2.212) 1.050(0.465-2.371)

***Health-related***

**Self-rated health status**

Good 1

Moderate 1.372(0.560-3.363)

Bad 1.379(0.579-3.284)

**Having NCD**

Yes 1

No 1.578(0.590-4.222)

**Model fitting information**

Number of observations 313 313 313 312 312

LR chi2 (7) 22.82 31.21 32.46 44.60 45.85

Prob > chi2 0.0018 0.0082 0.0132 0.0020 0.0046

Pseudo R2 0.0793 0.1084 0.1127 0.1568 0.1613

Log likelihood = -132.52974 -128.33189 -127.71037 -119.87537 -119.24602

*Source*: Computed from author’s survey data, September 2017 – October 2017

Asterisk values indicate significance of p value (p < 0.05), CI = confidence interval; AOR = adjusted odd ratio * p < 0.05

Model 1 = Socio-demographic variables; Model 2 = All variables in Model 1 plus socio‑economic variables; Model 3 = All variables in Model 2 plus Lifestyle risk factors variables; Model 4 = All variables in Model 3 plus living arrangement variables & Model 5 = All variables in Model 4 plus health-related variables
